# Validity of the Adapted Waterlow Score as a Tool in Predicting Adverse Outcomes in Acute Pancreatitis When Compared With the Ranson Score and Serum CRP Levels

**DOI:** 10.7759/cureus.25908

**Published:** 2022-06-13

**Authors:** Surya Prakash, Sameer Soni, Nikhil Tekwani

**Affiliations:** 1 Department of General Surgery, Medical College Baroda and Sir Sayajirao General Hospital, Vadodara, IND; 2 Department of Surgery, Chirayu Medical College and Hospital, Bhopal, IND

**Keywords:** acute pancreatitis, chronic pancreatitis, serum crp levels, waterlow score, ranson score

## Abstract

Introduction

Acute pancreatitis (AP) is one of the most common emergency room presentations, with an annual worldwide incidence of 150-420 cases per million and a 30-50% mortality rate. Unfortunately, predicting prognosis in AP patients at an early admission phase is difficult. The Waterlow score is a semi-quantitative composite score devised to stratify the risk of developing pressure sores in in-patient settings. Components such as age, gender, nutritional status, body mass index, mobility, smoking status, comorbidities, surgery, use of medications, neurological status, and continence, all combine to generate a single numerical figure between 2 and 64 to overcome the difficulty of the composite scoring systems such as the Ranson score, Glassgow score, Physiological and Operative Severity Score for the enUmeration of Mortality and morbidity (POSSUM) and its modification (Portsmouth or P-POSSUM), Simplified Acute Physiology Score (SAPS 2), Acute Physiology and Chronic Health Evaluation II (APACHE II) score, and Bedside index for severity in acute pancreatitis (BISAP).

Aim

This study aimed to validate the Waterlow score as a surrogate marker in predicting in-patient mortality, length of hospital stay (LOHS), intensive care unit (ICU) admissions, and AP complications compared to the Ranson score and serum CRP levels.

Method

A prospective case series analysis was conducted from August 2018 to November 2019 at a tertiary care center in central Gujarat. This study included 51 newly diagnosed patients with AP or acute on chronic pancreatitis on accrual. Out of 51 patients, four were excluded from our study, as three were discharged against medical advice (DAMA) and one other absconded from the ward premises. The study group comprised 47 patients diagnosed with AP, out of which 40 patients were male (85.1%) and seven were females (14.9%). The mean age of the population was 37.5 ± 13.0 years. Microsoft Excel calculated the standard deviation and mean for data entry. All statistical analyses were made with MedCalc software version 19.1.3 (MedCalc Software Ltd., Ostend, Belgium).

Result

When the Waterlow score, Ranson score, and serum C-reactive protein (CRP) levels were plotted against inpatient mortality, the area under the curve (AUC) was found to be 0.85, 0.80, and 0.98, respectively. The plot of CRP lay closest to the vertical axis. On plotting the receiver operator characteristic (ROC) curve of all three markers of AP severity, the area under the graph was calculated to be 0.81 (Waterlow score), 0.81 (Ranson score), and 0.82 (serum CRP), respectively. Nearly all three markers performed similarly. Waterlow score offers a high sensitivity but poor specificity in sorting outpatients at risk of adverse outcomes. It was observed that the Waterlow score could significantly predict mortality, ICU admissions, and LOHS in this study population. In contrast, the Ranson score and serum CRP levels could not indicate LOHS. A risk assessment tool based upon a subset of Waterlow parameters with additional variables may perform better than a tool based solely on Waterlow risk factors.

Conclusion

This study showed no correlation between the Waterlow score, the rate of local complications (p = 0.9513), and the incidences of surgical interventions (p = 0.9915). Therefore, a risk assessment tool based upon a subset of Waterlow parameters with additional variables may perform better than a tool based solely on Waterlow risk factors.

## Introduction

Acute pancreatitis (AP) is a common emergency room presentation, with a mortality rate of 30-50% [1]. The natural history of AP ranges from mild to severe forms. Though the etiopathology, diagnosis, and management of AP are now well-documented, the course of the disease process and its outcome remains unpredictable. Foretelling the severity and outcome of AP helps focus the healthcare resources on a particular subset of patients, thus providing optimum care [2]. At present, there is no established single reliable parameter that can predict the severity of AP. Markers deployed for predicting outcomes and studied extensively include C-reactive protein (CRP), melatonin, procalcitonin, glomerular filtration rate, arterial pH, base deficit, blood urea nitrogen, and hematocrit [3]. However, the results are not conclusive or reliable, and none of them have proven to be reliable predictive markers for estimating disease severity. To overcome the difficulty of the composite scoring systems, such as the Ranson score, Glassgow score, Physiological and Operative Severity Score for the enUmeration of Mortality and morbidity (POSSUM) and its modification (Portsmouth or P-POSSUM), Simplified Acute Physiology Score (SAPS 2), and Acute Physiology and Chronic Health Evaluation II (APACHE II) score, Bedside index for severity in acute pancreatitis (BISAP), Sequential Organ Failure Assessment (SOFA) Score, and Harmless Acute Pancreatitis Score (HAPS) have been formulated for diagnosing the severity of AP [4]. However, these tools are complex, contain lots of variables, and are based on multiple laboratory parameters and physiological scoring systems, which are expensive and cumbersome to calculate and implement in acute settings. They are also clinically ineffective in the presence of other patient-related pre-existent extraneous factors. Therefore, it would be beneficial to use an uncomplicated technique that could be readily measured in hospitals early on as part of the admission process for assessing the severity of AP and reliable prediction of adverse outcomes be made.

Waterlow score is a semi-quantitative composite screening tool introduced in the mid-1980s in many hospitals in the United Kingdom to stratify the risk of development of pressure sores in inpatient settings. The following components, such as age, gender, nutritional status, body mass index, mobility, smoking status, comorbidities, surgery, use of medications, neurological status, and continence [5], all combined to generate a single numerical figure between 2 and 64. Patients with scores over 10 were considered at risk of pressure ulcers while scores of over 15 and 20 represent high risk and very high-risk status, respectively. Its primary purpose was to focus on education, intervention, and resource management in the prevention of decubitus ulcers; however, it has been validated as an effective tool for predicting outcomes in other medical conditions, including AP and acute surgical emergencies [6].

## Materials and methods

Patient and methods

The present study assesses the Waterlow score compared to Ranson scoring and serum CRP levels in acute pancreatitis. It analyzes the comparison between these markers in the prediction of mortality, organ failure (OF), pancreatic necrosis (Pnec), length of hospital stay (LOHS), complications, operations, and ICU admissions. The study was conducted from August 2018 to November 2019 at Sir Sayajirao General Hospital, Baroda. It was a prospective observational cohort study. A total of 51 consecutive diagnosed patients with acute pancreatitis were included in this study. The present study included all patients who were newly diagnosed cases with acute pancreatitis. It also included attacks of acute pancreatitis in patients pre-existing. Patients who were unwilling to consent to blood investigations and were not included in the study, pregnant patients, and patients under 14 years of age were considered in the exclusion criteria.

The institutional ethics committee approved the study for human research, IECHR, Medical College & SSG Hospital, Baroda, dated 24/08/2018 (EC Reg No: ECR/85/Inst/GJ/2013/RR-16).

Out of 51 patients, four were excluded from our study, as three were discharged against medical advice (DAMA), and one other absconded from the ward premises. The study group comprised 47 patients diagnosed with AP, out of which 40 patients were male (85.1%) and seven were females (14.9%) (Table 1). The mean age of the population was 37.5 ± 13.0 years.

**Table 1:** Showing distribution of male and female sex in the study population

	Number of patients	Percentage %
Males	40	85.1
Females	7	14.9
Total	47	100

Methods

Informed written consent was obtained from all the patients. Diagnosis of AP was based on the following three features, abdominal pain typical of AP, serum amylase, and lipase ≥3 times the upper limit of average values and characteristic findings of AP on the abdominal radiological scan.

Demographic, radiographic, and laboratory parameters were acquired from all these patients. The Waterlow score was recorded within the first 24 hours of admission, the Ranson score was assessed at admission and after 48 hours of admission, and s.CRP values of these patients were recorded at 48 hours of admission. On day two, 2 mL of blood sample was collected for CRP. Arterial blood gas analysis (ABGA) analysis was done using the cartridge-based Abbott i-STAT handheld wireless system (Abbott Laboratories, Chicago, Illinois). CECT has been performed as and when required to look for local complications and possible etiology of AP.

Continuous variables were expressed as means. Microsoft Excel was used to calculate the standard deviations and mean for data entry. Each scoring tool was divided into subsets, Waterlow scores into ≤10, 11-15, 16-20, and ≥20. Ranson scores were segregated into 0, 1, 2 and ≥3, and serum CRP values into <10 mg/dL, 10-100 mg/dL, 100-150 mg/dL, and above 150 mg/dL. Each predictive parameter was evaluated at the selected cut-off scores for the significant relationship to the mortality, LOHS, ICU admissions, and complications. The area under the receiver-operator curves measured the predictive accuracy of each scoring system and the biochemical marker. All statistical analyses were made with MedCalc software version 19.1.3 (MedCalc Software Ltd., Ostend, Belgium).

## Results

Fifty-one patients diagnosed with acute pancreatitis satisfying the inclusion and exclusion criteria were enrolled in the present study. Out of them, four patients were excluded from the study, as three were discharged against medical advice (DAMA) and one other absconded from the ward. These patients were subjected to a risk assessment by evaluating their Waterlow scores within 24 hours of hospital admission. Simultaneously, Ranson's score and serum CRP levels were also recorded.

Etiology

Alcoholism was the cause of AP in most male patients (35 out of 40 patients, 87.5%). On the other hand, gallstone was the commonest etiology observed in the female gender (4 out of 7 patients, 57.1%). Autoimmune and hypercalcemia as the etiology of AP contributed a share of less than 5% (Table 2).

**Table 2:** Showing etiologies and their relative incidences

Etiology	No of males	% males	No. of females	% females	Total patients	%
Alcohol-induced	35	87.5	2	28.57	37	78.72
Gallstone	4	10	4	57.14	8	17.02
Autoimmune	1	2.5	0	0.00	1	2.13
Idiopathic	0	0	1	14.29	1	2.13
	40	100	7	100.00	47	100.00

Out of 47 patients, 36 (76.6%) of them developed mildly severe AP, six patients (12.8%) with moderately severe AP, and four patients (8.5%) with severe AP. As per the determinant-based classification, one of the patients (2.13%) in the study sample had critical AP (Table 3).

**Table 3:** Showing distribution of severity of AP patients AP: acute pancreatitis

Severity of AP	No. of patients	Percentage
Mild	36	76.60
Moderate	6	12.77
Severe	4	8.51
Critical	1	2.13
Total	47	100

Inpatient mortality

Four patients succumbed due to the disease process in the sample of 47 patients diagnosed with acute pancreatitis. The overall incidence of mortality was 8.51%. As the Waterlow score exceeded 20, mortality rates were highest (66.7%), with two out of three patients dying of the disease while mortality in patients who scored below 15 was less than 5%. An increasing trend was observed concerning the mortality rate, increasing the Waterlow score (Table 4).

**Table 4:** Inpatient mortality

Waterlow score	Number of patients	Mortality	Mortality rate (%)
≤10	19	0	0
11 to 15	21	1	4.76
16 to 20	4	1	25.00
>20	3	2	66.67
Total	47	4	-

Three out of 5 patients (60%) with a Ranson score of 3 or more died due to the disease process while deaths among patients with a Ranson score of 2 or less were lower than 5% (Table 5).

**Table 5:** Ranson score distribution and incidence of deaths

Ranson score	Number of patients	Mortality	Mortality rate (%)
0	19	1	5.26
1	14	0	0
2	9	0	0
≥3	5	3	60.00
Total	47	4	-

The incidence of mortality with serum CRP levels also followed a similar pattern. All patients with serum CRP values higher than 150 mg/L died from the disease while there was no mortality observed when CRP levels remained below 100 mg/L (Table 6).

**Table 6:** Mortality in different CRP subsets CRP: C-reactive protein

s. CRP level (mg/L)	Number of patients	Mortality	Mortality rate (%)
0-50	30	0	0
50-100	10	0	0
100-150	5	2	40
>150	2	2	100
Total	47	4	-

Length of hospital stays (LOHS)

The length of hospital stays followed a standard distribution curve, i.e., the bell-shaped curve, except for the outlier value, which was the admission of 56 days in which the patient was operated upon. Length of hospital stays was lower on either extremity of the Waterlow score. Among the patients with higher Waterlow scores (i.e.,>20), mortality rates were high, and they expired at an early phase of hospital stay while the patients who had scored less than 10 were inclined towards chronicity with a milder form of the disease. They eventually were discharged earlier, with a shorter hospital stay. Nine out of 10 patients operated on had their Waterlow scores ranging from 11-20. As they were operated, they had a longer course of the disease and thus required more prolonged hospital admissions (Table 7).

**Table 7:** Length of stay according to the Waterlow score

Waterlow score	Length of stay (mean±SD)
≤10	7.1± 4.0 days
11 to 15	14.9± 12.6 days
16 to 20	12.3±6.6 days
˃20	8.0±4.4 days

ICU admissions

Seven out of 47 patients (14.89%) were admitted to the ICU care setup. The mean ICU stay was 2.7 ± 1.1 days, with a median of three days. Patients with greater Waterlow scores were often frequently admitted into ICU setups. In the present study, four out of seven patients (57.1%), having scored more than 15, were shifted to intensive care units, compared to nil ICU admissions when the score remained below 10.

Local and systemic complications

**Figure 1:**
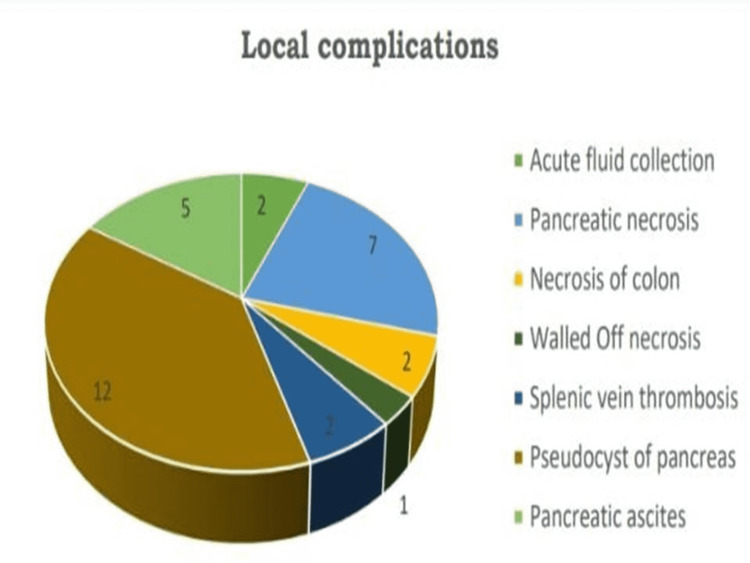
Local complications

Twenty-five out of 47 patients did develop local complications. However, some developed a single complication (19 out of 25, 76%) while others developed multiple local complications (6 out of 25, 24%) (Figure 1).

Organ failure occurred in six patients. Out of which, single organ failure occurred in four patients (66.67%), and two patients developed multiple organ failure (33.33%). Renal failure was the most common (8.51%) while cardiovascular failure was the least common type of OF met (2.13%). OF was strongly related to the incidences of mortality in AP patients (chi-square=9.6, p-value=0.001) (Table 8).

**Table 8:** Showing percentage of different organ failures

Organ failure (OF)	No. of cases	Percentage
Renal Failure	4	8.51
Hepatic	3	6.38
Cardiovascular	1	2.13

Surgical interventions

Ten out of 47 patients required some surgical intervention. Surgeries were done in acute presentations to control sepsis, such as external drainage of infected pancreatic pseudocyst by open method (1 patient), necrosectomy (2 patients), incision & drainage of retroperitoneal abscess (1 patient), and minimally invasive procedures like insertion of a Malecot catheter under ultrasound (USG) guidance into the cyst cavity (2 patients). Definitive surgeries to control the etiology of the disease and its complications were needed for four patients. Cholecystectomy was done (in case of gallstone-induced pancreatitis) in two patients; one patient required cystogastrostomy (in case of pancreatic pseudocyst) while exploratory laparotomy with the division of gastroduodenal artery was done (in case of hemorrhagic pancreatic pseudocyst with an aneurysm) in one of the patients.

Patient factors

When the Waterlow score, Ranson score, and serum CRP levels were plotted against inpatient mortality, the AUC was found to be 0.80 and 0.98, respectively. The plot of CRP lay closest to the vertical axis. On plotting the receiver operator characteristic curve of all three markers of AP severity, the area under the graph was calculated to be 0.81 (Waterlow score), 0.81 (Ranson score), and 0.82 (serum CRP), respectively. Nearly all three markers performed similarly.

Waterlow score offers a high sensitivity but poor specificity in sorting outpatients at risk of adverse outcomes. It can be interpreted as patients with a high Waterlow score are segregated as being "at-risk", but not all of the "at-risk" population will develop the adverse outcome, as dictated by the poor specificity of the score. In other words, the Waterlow score overestimates the subject's mortality risk in AP patients.

Mortality rate in AP patients was statistically insignificant with different BMI values (chi-square=1.24, p-value=0.26). Anemia (with cut-off taken at Hb value <8 g/dL) at the time of hospital admission was not related to mortality in cases of AP (chi-square=0.79, p-value=0.37).

## Discussion

Acute pancreatitis is a significant surgical burden on hospital resources. The present research was a prospective study conducted at a single tertiary care center, Sir Sayajirao General Hospital, Vadodara, Gujarat, to look for the reliability of the Waterlow score in predicting mortality in patients diagnosed with acute pancreatitis. Fifty-one patients were enrolled in the study and four were excluded, as they were discharged against medical advice or absconded from the ward.

The present study assessed patients for their Waterlow and Ranson scores and their s.CRP levels. Mortality, length of hospital stays, ICU admissions, and complications were recorded. In addition, the Association of Waterlow score with these parameters was evaluated. In the present study, more than half of the sample comprised adult males 30-50 years of age. On the contrary, the younger and older population contributed significantly less to the disease. The argument can justify this that most people start the habit of alcohol consumption at around 20-25 years of age, and the onset of alcoholic pancreatitis occurs in the fourth decade of life. Hence, when the same population attains an adult age, incidences of AP are observed at their peak.

The mean age of the present study group was 37.5 ± 12.97 years. A similar profile of age distribution was observed in other studies. Deherkar JA et al. surveyed 100 patients with AP. The mean age of the study population in this group was 38.3 ± 12.92 years, which was analogous to the present study [7]. Like this, as studied by Sharma V et al., the age demography also depicted the mean age as 39.33 ± 13.85 years (range 13-75 years) [8].

The present study group comprised 47 patients diagnosed with acute pancreatitis, out of which 40 were male (85.1%) and seven were females (14.9%). A higher incidence of AP was seen in the male gender. It can be attributed to the most common cause of pancreatitis, i.e., alcoholism is still widely seen in males. Consumption of alcohol is considered a social stigma for females in India. Hence, the female population presenting with AP was very meager. Akin to the sex distribution of the present study, in other studies as done by Tee Yu-San et al., Gooptu S et al., and Park JY et al., the contribution of the male sex in the disease was more than 70% [9-11].

The research done by Khanna A et al. portrayed a lower male preponderance of the disease (51.4% and 61.0%, respectively) [12]. Therefore, it can be explained that the biliary etiology of AP is more commonly seen in North Indian females of India, and both of these studies were done in North India. Alcoholism remains the leading cause behind the etiology of acute pancreatitis. In the present study, alcohol was the commonest etiology in 37 out of 47 patients, with 78.7%. The high prevalence of consumption of alcohol still overshadows the other etiologies behind the genesis of AP in western parts of India.

Gallstone remained the next major cause, with eight people affected by gallstone-induced AP. (17.02%) More commonly, this was seen in the female sex. (4 out of 7 patients, 57.1%). Stirling AD et al. showed a parallel distribution of different etiologies where alcoholic pancreatitis remained the primary etiology (49.8%, 46.6%, and 43.7%, respectively) [13].

In the present study, four patients could not survive and died of the disease. Therefore, the rate of mortality was 8.5%. Nearly identical results concerning mortality rate were observed in studies done by Khanna KA et al. and Gillick K et al. (12.5% and 8.0%, respectively) [1,12]. In the study by Halonen KI et al., hospital mortality in severe acute pancreatitis in a general intensive care unit was as high as 38.1% [14]. It can be explained, as the inclusion criteria of the study sample consisted of patients who were admitted to the ICU and had severe acute pancreatitis.

The length of hospital stay was lower on either extremity of the Waterlow score. The average LOHS was 7.1 ± 4 days for a Waterlow score of less than 10. While for scores >20, the mean LOHS was 8 ± 4.4 days. The patients with higher Waterlow scores' (i.e.,>20) mortality rates were high and they expired at an early phase of hospital stay, contributing to an overall lower mean length of stay while patients who had scored less than 10 had had a milder form of the disease. They eventually were discharged earlier, with a shorter hospital stay.

The median hospital stay length was eight days in the present study. However, the duration of median length of hospital stays in other studies as carried out by Roberts S E et al., Gillick K et al., Park JY et al., and Khanna KA et al. was similar to the present study [1,11-12,15]. The present study's overall admission rate into an ICU setting was 14.89% (7 out of 47 patients). When Waterlow scores exceeded 15, the patients had a rate of 57.1% (4 out of 7 patients), and these patients needed high dependency care in the ICU setup. The rate of ICU admission was similar to that of research done by Khanna K et al. [12].

Strengths of the study

Very few studies in the past have related to the potential value of the Waterlow score as a marker for screening the severity of AP patients who will eventually require more attention and intensive management due to the possibility of poorer outcomes in these surgical patients. This was a prospective observational cohort study where the relationship of patient factors, such as BMI and anemia, was studied as an adjunct factor behind mortality in AP patients, as well as their co-relations with Waterlow scores.

Limitations

Few components of the Waterlow scoring system carry an iota of subjectivity. Some less-agreed parameters are mobility, skin condition, and neurological deficits. The degree of neurological deficit is not clearly defined in the scoring pattern. Being operator-dependent, these components inherit the risk of decreased inter-rater (and even intra-rater) reliability. It limits the tool's applicability for a broader application in assessing the risk or severity of the disease. Multicentric, more extensive studies need to be conducted to understand this score's utility better. The reliability of the tool can only be attained by education and its usage over a period of time. Furthermore, the sample size of the present study was small, and the study was carried out at a single tertiary care center. More such studies need to be carried out at a broader, multicentric level, with larger sample size, for further comments.

Future implications

A risk assessment tool based upon a subset of Waterlow parameters, with additional variables, such as CRP levels, may perform better than a tool based solely on Waterlow risk factors. This tool should not be used as the sole gauge for allocation of resources but as an aid in conjunction with clinical judgment and other biochemical parameters.

## Conclusions

The present study showed that acute pancreatitis manifests mainly in young adult males, 30 to 50 years. Alcohol was the most common factor to be blamed for the etiology behind the disease. Gallstone was the second most common etiology. Mortality in patients with AP lies around 8.5%. The observed deaths increased with Waterlow, Ranson, and serum CRP levels. The Waterlow score calculated at the time of hospital admission proved an efficient tool for predicting inpatient mortality, length of hospital stays, and need for ICU admissions. Ranson's score seemed to be an equally good indicator of mortality in AP. The Waterlow score failed to significantly predict the development of complications and the need for surgical interventions. Patient factors, such as anemia and BMI, were not related to incidences of mortality in patients with AP. Systemic complications like organ failure were intimately linked with deaths in acute pancreatitis. Some of the Waterlow score parameters, such as special risk categories, including a neurological deficit, surgery/trauma, and medications, remained irrelevant for patients of AP. None of the study subjects fell under any of the above-mentioned categories. Ninety-five percent (95%) of the study population had serum CRP values less than 150 mg/L. Usually, patients of the present study group were those who were referred from other smaller medical units in rural or suburban areas. It resulted in delayed presentation of the above set of patients due to which their accurate values could not be evaluated. Serum CRP values peak at 48 hrs of the onset of symptoms and decreases afterward. It might have been possible that this data could have been different if estimated at the proper time interval.
